# Occult posterolateral rotatory dislocation of the elbow with olecranon fracture in a child: a case report

**DOI:** 10.1186/1752-1947-6-273

**Published:** 2012-09-03

**Authors:** Takahito Fujimori, Kohji Kuriyama, Koji Yamamoto, Hisao Moritomo, Hideki Yoshikawa

**Affiliations:** 1 Department of Orthopedic Surgery, Osaka University Graduate School of Medicine, 2-2 Yamadaoka, Suita, Osaka, 565-0871, Japan; 2 Department of Orthopedic Surgery, Hoshigaoka Koseinenkin Hospital, 4-8-1 Hoshigaoka, Hirakata, Osaka, 573-8511, Japan; 3 Department of Orthopedic Surgery, Toyonaka Municipal Hospital, 4-14-1 Shibahara, Toyonaka, Osaka, 560-0055, Japan

## Abstract

**Introduction:**

Acute posterolateral rotator elbow dislocation in a child is rare and can be easily misdiagnosed due to immaturity of the epiphysis. This is the first case of occult posterolateral rotator elbow dislocation in combination with an olecranon fracture. We report our experience with this case, which was not diagnosed correctly by plain radiographs.

**Case presentation:**

An 11-year-old Asian boy suffered severe pain and swelling of his right elbow after his outstretched arm hit a car dashboard in a motor vehicle accident. Plain radiographs showed only a minimally displaced olecranon fracture and a tiny lateral epicondylar avulsion fracture. However, stress radiographs under general anesthesia revealed severe posterolateral rotatory instability. During surgery, we found that the cartilaginous lateral epicondylar apophysis was much larger than the epicondylar fragment on the radiographs. After the lateral epicondylar osteochondral fragment and lateral collateral ligament complex were fixed, the instability disappeared.

**Conclusion:**

Our experience with this case shows that it is important to check for instability with pediatric elbow fractures, because a tiny avulsion fracture was able to cause severe posterolateral rotatory instability in a child.

## Introduction

Acute posterolateral rotator elbow dislocation in a child is rare and can be easily misdiagnosed due to immaturity of the epiphysis. We report what is, to the best of our knowledge, the first case of occult posterolateral rotator elbow dislocation in combination with an olecranon fracture, which occurred in a child who was not diagnosed correctly by plain radiographs. We show that an instability test leads to a correct diagnosis and early repair can prevent chronic ligament instability and nonunion.

## Case presentation

**Figure 1:**
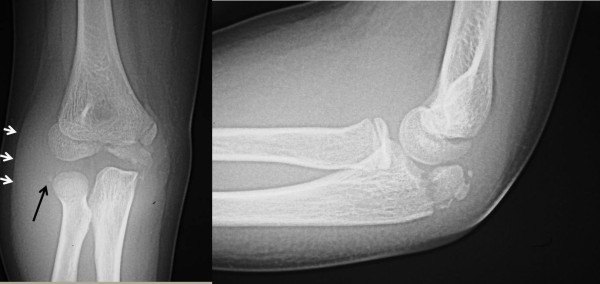
**Anteroposterior and lateral radiographs of the elbow.** (**A**) Anteroposterior radiograph showing a small avulsion of the lateral epicondylar fragment (long arrow) and soft-tissue swelling (short arrows). (**B**) Lateral radiograph showing the olecranon fracture.

**Figure 2:**
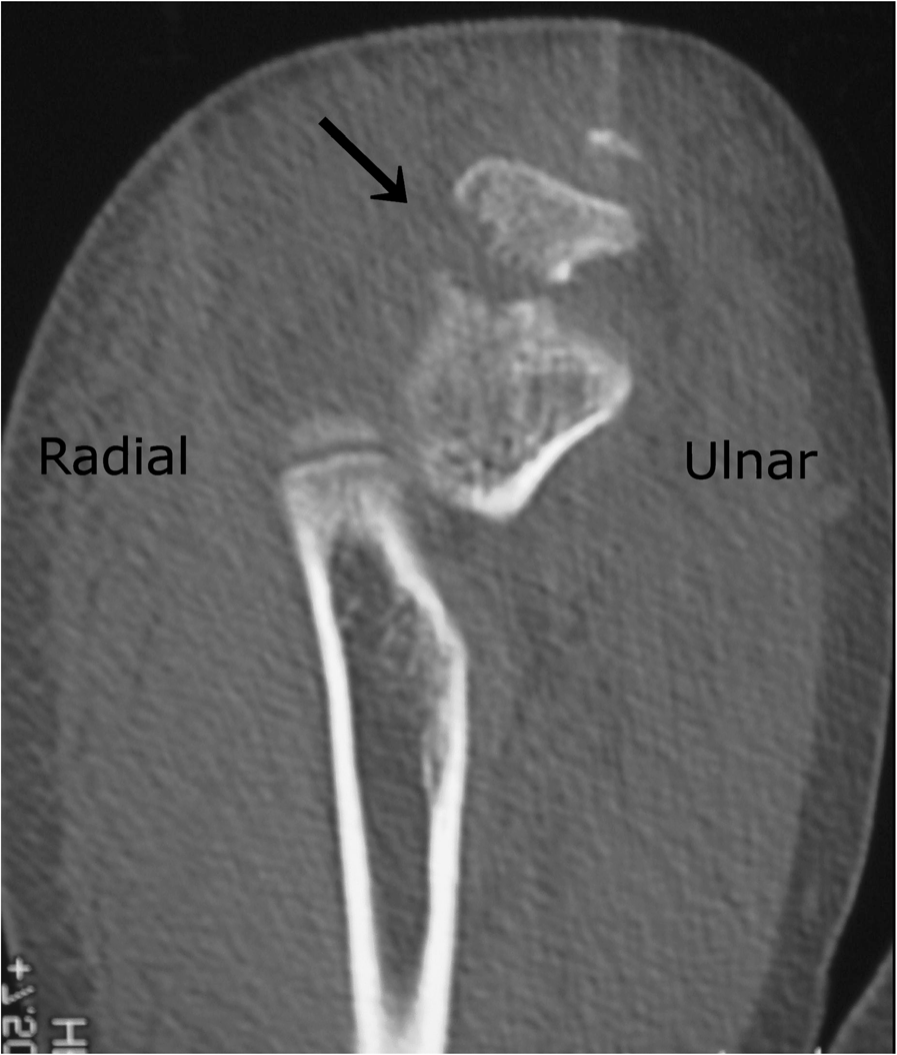
Computed tomography scan of the elbow showing the olecranon fracture line (arrow) running from proximal radius to distal ulnar.

An 11-year-old boy arrived at our institution’s emergency department after his outstretched arm hit a car dashboard in a motor vehicle accident. Our patient reported severe pain, and his elbow was swollen and had a limited range of motion. Findings on a neurovascular examination were normal. His past medical history was not significant, with no previous elbow injuries. An anteroposterior radiograph showed a small lateral epicondylar avulsion fracture and considerable soft-tissue swelling, and a lateral radiograph showed an olecranon fracture (Figure 1). To exclude other possible complicated fractures, computed tomography (CT) was performed. CT revealed an oblique olecranon fracture running from his proximal radius to distal ulnar (Figure 2). The proximal radioulnar joint of his elbow was intact and there was no Monteggia equivalent type fracture.

**Figure 3:**
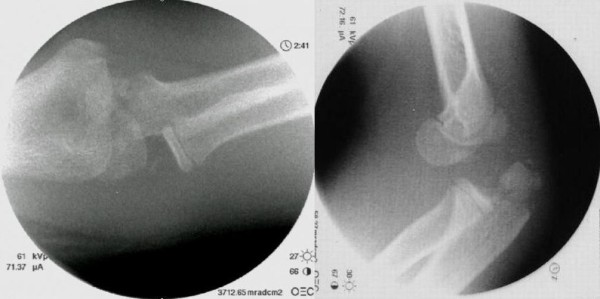
**Image intensifier of instability test.** (**A**) Varus stress image showing enlargement of the radiohumeral joint space, suggesting insufficiency of the lateral collateral ligament. (**B**) The elbow was easily redislocated with the forearm placed in a supine position.

After induction of general anesthesia, dynamic images were obtained and surgery was performed. A varus stress image showed enlargement of the radiohumeral joint space, suggesting insufficiency of the lateral collateral ligamentous stabilizer (See Additional file 1: varus instability). There was no valgus instability. The posterolateral rotatory instability test easily reproduced posterolateral rotatory elbow dislocation (Figure 3). When the elbow was flexed more than 50°, the elbow joint was reduced with a palpable clunk (See Additional file 2: posterolateral rotator instability).

**Figure 4:**
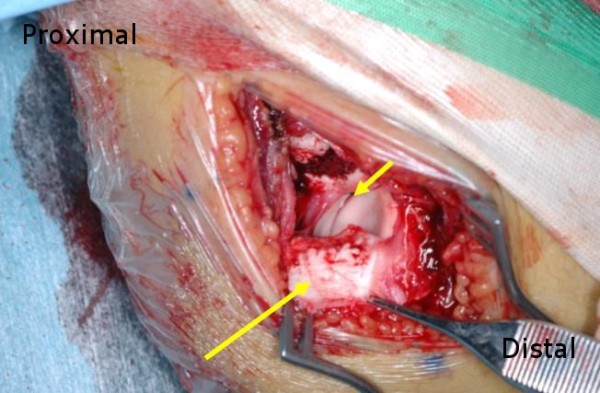
**The surgical field from a lateral approach.** The lateral collateral ligament complex was avulsed with a large lateral epicondylar osteochondral fragment (long arrow). The proximal radioulnar joint was intact (short arrow).

**Figure 5:**
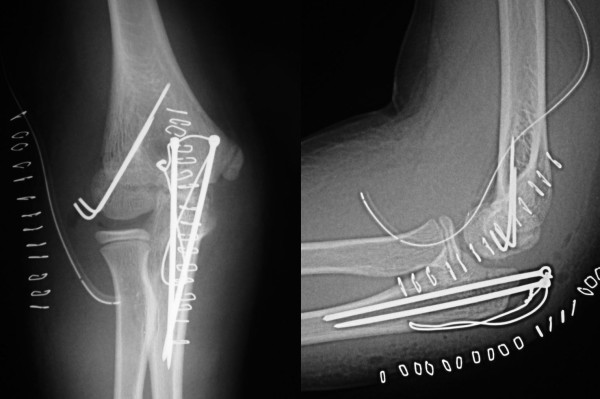
**Postoperative anteroposterior (A) and lateral (B) radiographs of the elbow**.

First, we fixed the olecranon fracture using a tension-band wiring method from a posterior approach. However, his elbow still had gross posterolateral rotatory instability, so we used a lateral approach to repair the lateral stabilizer. The lateral capsule was ruptured at the proximal attachment, and there was a large hematoma. The cartilaginous lateral epicondylar apophysis by which the lateral collateral ligament complex (LCLC) was attached had avulsed with a lateral condylar fragment (Figure 4). The cartilaginous lateral epicondylar apophysis was much larger than the epicondylar fragment seen on the radiographs. A part of the common extensor origin was also disrupted. His annular ligament remained intact. After we performed reduction and fixation of the cartilaginous lateral epicondylar fragment with Kirschner wires, the instability disappeared (Figure 5).

His arm was immobilized in a splint with his elbow flexed at 90° and his forearm at 30° of pronation. Three weeks after surgery, the splint was removed and active exercise of his elbow was started. Six months after surgery, radiographs showed bony union, and there was no instability. The Kirschner wires were removed in a second operation. Two years after the first surgery, our patient had neither pain nor subjective instability, and his elbow had a nearly full range of motion: flexion, 135°; extension, 0°; pronation, 80°; supination, 90°.

## Discussion

O’Driscoll *et al*. [1] have previously described chronic posterolateral rotatory instability due to insufficiency of the lateral ulnar collateral ligament in which a valgus, axial and supination force was a provoking test that induced posterolateral rotatory dislocation. Acute posterolateral rotatory dislocation of the elbow joint is rare; to the best of our knowledge only one case had been reported before ours: Imatani *et al*. reported the case of a 60-year-old man with acute posterolateral rotatory dislocation due to LCLC insufficiency caused by varus stress [2]. Only three cases of posterior elbow dislocation with lateral condylar avulsion fracture have been reported [3-5]. Van Haaren *et al*. [5] reported such a case involving a six-year-old girl. They suggested that a varus force induced the injury, noted the risk of subsequent dislocation, and recommended prompt open reduction and fixation. Rovinsky *et al*. [3] reported the case of an 11-year-old boy with posterior dislocation of the elbow with a lateral condyle avulsion fracture. Although they palpated a large lateral fragment in their patient, it only appeared as a small fragment on radiographs. Thus, they emphasized the need for careful physical examination for making a correct diagnosis. The mechanism of injury was reported to be varus stress applied to the extended elbow with the forearm supinated. Although none of these reports referred to the posterolateral rotator instability test, we consider these cases to have been acute posterolateral rotator dislocations. To the best of our knowledge, there are no reports, other than ours, of acute posterolateral rotator elbow dislocation with an olecranon fracture and a lateral epicondyle avulsion fracture in a child.

Generally, the elbow is likely to be affected by valgus stress because of the physiological cubitus valgus. However, as previous reports have noted, the mechanism of these injuries was believed to be varus stress on a fully extended elbow with a supinated forearm. The direction of the fracture line in the olecranon detected by CT, running from the proximal radius to the distal ulnar, confirms that varus stress was placed on the olecranon. In this case, CT was useful for evaluating these complicated fractures. Magnetic resonance imaging might be another choice of diagnosis method, considering radiation exposure.

It is sometimes hard to differentiate a normal ossification center from an avulsion fracture because the ossification center is separated from the lateral condylar epiphysis. Generally, the ossification center of the lateral epicondyle can be detected on radiographs by the time a patient is 10 years of age; the ossification process starts at the exterior of the epicondyle and moves to its center. Silberstein *et al*. [6] reported the detailed ossification process of the lateral epicondyle and emphasized soft-tissue swelling detectable on radiographs as important in the differential diagnosis of lateral epicondylar fractures. Careful interpretation of radiographs is important because the epicondylar apophysis commonly accompanies a cartilaginous fragment sliver larger than might be expected on radiographs. Most recurrent elbow dislocations in adults are thought to arise because the initial dislocation occurred before skeletal maturity. Osborne and Cotterill [7] indicated that a pocket in the lateral collateral ligament with a nonunited lateral epicondylar fragment could cause recurrent elbow dislocation and instability.

## Conclusion

Our case illustrates that early initial repair of the LCLC and olecranon fracture, after careful physical examination of LCLC insufficiency and interpretation of radiographs to make a correct diagnosis, can produce excellent results. We recommend physicians to check for instability with a pediatric elbow fracture because, in our case, a tiny avulsion fracture was able to cause instability in a child.

## Consent

Written informed consent was obtained from the patient and the patient’s parents for publication of this case report and any accompanying images. A copy of the written consent is available for review by the Editor-in-Chief of this journal.

## Competing interests

The authors declare that they have no competing interests.

## Authors’ contributions

TF was involved in all aspects of the case report, from data collection, writing the manuscript, and editing to final approval. KK, KY and HM strictly reviewed the article. HY made the final review. All authors read and approved the final manuscript.

## Supplementary Material

Additional file 1 The movie showed that there was varus instability. Click here for file

Additional file 2 The movie showed that there was posterolateral rotatory instability. Click here for file
